# The effects of socioeconomic status and indices of physical environment on reduced birth weight and preterm births in Eastern Massachusetts

**DOI:** 10.1186/1476-069X-7-60

**Published:** 2008-11-25

**Authors:** Ariana Zeka, Steve J Melly, Joel Schwartz

**Affiliations:** 1 Department of Environmental Health, Harvard School of Public Health, Harvard University, Landmark Suite 415 West, Boston, MA 02115, USA; 2 Institute for the Environment, Brunel University West London, UB8 3PH, UK

## Abstract

**Background:**

Air pollution and social characteristics have been shown to affect indicators of health. While use of spatial methods to estimate exposure to air pollution has increased the power to detect effects, questions have been raised about potential for confounding by social factors.

**Methods:**

A study of singleton births in Eastern Massachusetts was conducted between 1996 and 2002 to examine the association between indicators of traffic, land use, individual and area-based socioeconomic measures (SEM), and birth outcomes (*birth weight, small for gestational age *and *preterm births*), in a two-level hierarchical model.

**Results:**

We found effects of both individual (education, race, prenatal care index) and area-based (median household income) SEM with all birth outcomes. The associations for traffic and land use variables were mainly seen with birth weight, with an exception for an effect of cumulative traffic density on *small for gestational age*. Race/ethnicity of mother was an important predictor of birth outcomes and a strong confounder for both area-based SEM and indices of physical environment. The effects of traffic and land use differed by level of education and median household income.

**Conclusion:**

Overall, the findings of the study suggested greater likelihood of *reduced birth weight *and *preterm birth*s among the more socially disadvantaged, and a greater risk of *reduced birth weight *associated with traffic exposures. Results revealed the importance of controlling simultaneously for SEM and environmental exposures as the way to better understand determinants of health.

## Introduction

Adverse birth outcomes such as low birth weight and its determinants, preterm births and intrauterine growth retardation have been associated with indicators of socioeconomic status and physical environment [1-3]. The issue stands high in the public health concerns due to the evidence that adverse health in early life can lead to later life diseases of childhood and adulthood [4-10].

Socioeconomic status is a determinant of health in populations [11,12], and it has been linked to adverse birth outcomes [3,13-16]. It is likely that socioeconomic status is a measure of access to health care, empowerment, level of stress and violence, and likelihood of exposure to environmental factors, and among these air pollution exposure [17,18]. Air pollution, a component of the physical environment, has also been associated with low birth weight and preterm births [19-24]. While different sources of air pollution have been associated with increased risk of adverse health, it is traffic pollutants that show the greater heterogeneity in concentration within urban areas [25]. Because measures of concentrations of air pollutants at individual addresses are unfeasible in large studies of births or other outcomes, either modeled concentrations of air pollutants or measures of traffic such as traffic density or distance to roadways have been used as exposure indices [26]. One concern of this approach is that variability in socioeconomic measures (SEM) can predict distribution of indices of physical environment [27-30]. Hence by studying these two together one can control each for confounding by the other, also allowing for examination of interactions between factors. In addition, the use of SEM at both the individual and area-based level allows one to capture both the effect of individual level SEM as well as any additional contextual effects that exist at the area level. Most epidemiologic studies of pregnancy outcomes have examined the effects of individual or contextual level SEM or the effects of physical environment, but rarely the effects of all three simultaneously.

The present study was carried out to identify and explain disparities in reduced birth weight and preterm births in seven counties of Eastern Massachusetts between 1996 and 2002. We intended in this study to explain the variation in birth outcomes as a function of the individual and area-based level SEM, measures of traffic and land use as indices of physical environment, and their interaction using a two-level hierarchical model. We also sought to assess the degree of confounding of one group of factors by the other likely to be present in previous reports that have not examined these two groups of factors together.

## Methods

### Study population

The study population included *all singleton live births *in Eastern Massachusetts for the counties of Bristol, Essex, Middlesex, Norfolk, Plymouth, Suffolk, and Worcester obtained from the Massachusetts Birth Registry for the period between January 1, 1996 and December 31, 2002. The population of the seven counties covered about 83 percent of the state's population. Individual address of mother at time of birth was geocoded by a private firm by matching the address to state, city ZIPcode and TIGER street network data, and assigning to this street address latitude and longitude coordinates. The geocoding was then reassessed by us for accuracy and completeness. The study and the use of birth data was approved by the Massachusetts Department of Public Health and the Human Subjects Committee of the Harvard School of Public Health. We restricted our study to births born between 20 and 45 weeks of gestation, with birth weight between 500 and 5500 grams. We also excluded those births that could not be correctly assigned an address (4.9 percent), resulting in the final number used for the study of 425 751.

### Outcome

We focused on three measures of birth outcomes: *birth weight *(continuous variable), *small for gestational age *(SGA) and *preterm births *(dichotomous variables). Low birth weight among term newborns (born at ≥ 37 to 45 weeks of gestation) may represent intrauterine growth retardation. We constructed a measure of intrauterine growth retardation, SGA, by calculating the lowest 10 percentile of the distribution of weight by gestational week, maternal race, and infant gender among all term births as the cutoff. We assigned 1 to those births falling below this cutoff and 0 otherwise (reference group). *Preterm birth*s were defined as all neonates born between 20 and <37 weeks of gestation. The reference group for preterm births was defined as all term births (born at ≥ 37 to 45 weeks.

### Exposure

We used three indicators of physical environment for this study. We calculated two measures of traffic – cumulative traffic density and distance to primary highways, and assigned them at individual address of mother at time of birth. We also calculated the percent land used in each census tract for recreation and conservation.

#### Cumulative traffic density

We obtained a spatial dataset for roads and highways in Eastern Massachusetts in 2002 from the Massachusetts Highway Department (MHD 2002), which conducts annual traffic data collection. This dataset included average daily traffic (ADT) as an attribute.

A grid made up of points spaced 200 meters apart was created over the study area of Eastern Massachusetts, using ArcGIS 9.1 (ESRI, Redlands, CA) buffer tool. Around each grid point we created 100 meters radius circles. We intersected these circles with the MHD 2002 roads to create a combined dataset of roads within each circle. Cumulative ADT (CADT) was calculated for all road segments within 100 meters around each grid point as: CADT = Σ (ADT * road segment was assigned to the respective grid point. Birth addresses were then assigned a weighted average of the estimates of the four grid points around it, using bilinear interpolation.

#### Distance to roadways

Spatial data on major roadways were obtained through the US Census 2000 Topologically Integrated Geographic Encoding and Referencing system (TIGER) [31]. Geocoded birth addresses were joined to these data by spatial location, using ArcGIS 9.1. Using the resulting database we identified the nearest primary highway with "limited access" (these roads can only be accessed through ramps, have multiple lanes of traffic, and have no direct intersection with other roads) to the birth address and calculated the distance to this major road. The TIGER roads were chosen because the same database source was used for geocoding the birth addresses.

#### Land use for recreation and conservation (open space)

Protected and recreational open space was downloaded from MassGIS, Massachusetts Executive Office of Environmental Affairs [32]. The subset of the open space designated for recreation, conservation, water supply, and forestry was intersected with 2000 Census tract boundaries (also downloaded from MassGIS) using ArcGIS 9.1. The percent of each census tract that was open space was then calculated and assigned to birth addresses belonging to that tract.

### Socioeconomic indicators

#### Individual variables

We obtained from the birth registry information on mother's race, years of education, and the Kotelchuck index of adequacy of prenatal care utilization (APNCU). Race/ethnic background of mother was categorized as: White, African-American, Asian, and Hispanic. Due to small numbers we excluded births from Native American mothers from the analyses (total of 674).

We categorized education as: *high school or less *(≤ 12 years of educational attainment); *some college *(13–15 years); and *college or postgraduate *(≥ 16 years). We categorized the APNCU, a measure based on the number and the time of start of mother's prenatal visits [33], into: *inadequate *(<50 percent of expected visits used); *intermediate *(50–79 percent); *appropriate *(80–109 percent); and *appropriate plus *(≥ 110 percent). Adequacy of prenatal care can be a predictor of accessibility of mother to health care, and inability to have appropriate prenatal care is more likely for those living in poorer neighborhoods.

#### Contextual variables

We obtained data from the United States Census Bureau of 2000 on median household income for each census tract in the study area, and assigned these to births whose address belonged to that tract. Preliminary analyses controlled for several other variables at the census tract level: percent below poverty, percent with low education, percent of ethnic background (African-American, Hispanic) and found no association or confounding by these factors, therefore not presented in this work.

### Model covariates

Measures of traffic were log transformed to stabilize the variance and used as such in the analyses. We controlled additionally in the models for age of mother, gestational age, amount of cigarettes smoked during pregnancy, chronic conditions of mother or conditions of pregnancy (renal disease, lung disease, hypertension, gestational diabetes or diabetes diagnosed otherwise, uterine bleeding), if the mother ever had a previous preterm birth, if the mother ever had a previous infant weighting 4000 grams or more, gender of infant, and year of birth to control for time trends.

Smoking during pregnancy is associated with adverse births, and it has not been controlled for in many previous studies. Because smoking varies with social class in the United States [34], it may represent an important confounder. Information on smoking was self-reported by mother on birth certificate, and it reflects the number of cigarettes smoked per day during and pre-pregnancy.

### Statistical Methods

#### Modeling for gaussian data

For continuous outcomes such as birth weight, linear mixed regression models were carried out [35]. For example, let Y_ij _be the response (*birth weight*) in the i^th ^subject in census tract j, and child sex_i_, maternal age_i_, traffic at individual address of mother_i_, individual-level socioeconomic measures (ILSEM)_i_, census tract-level socioeconomic measures (TLSEM)_j _denote the set of covariates of interest. Then we considered models of the form

*Y*_*ij *_= *u*_*j *_+ *b*_0 _+ *b*_1_*child sex*_*i *_+ *b*_2_*maternal age*_*i *_+ ... + *b*_3_*Traffic*_*i *_+ *b*_4_*ILSEM*_*i *_+ *b*_5_*TLSEM*_*j *_+ ... + *e*_*ij*_

Here *e*_*ij *_is the error term, and u_j _is the tract-specific random intercept and represents the variation of the rate in the groups due to unmeasured factors. This intercept is randomly generated from a normal distribution as suggested by Pickle [36], modeled with the SAS procedure MIXED (SAS Institute).

#### Modeling for binomial data

Binomial data was modeled similarly to the normal data. For *SGA *and *preterm births*, Y_ij _will be the outcome in the i^th ^subject in the j^th ^census tract, with the model of the form

Logit(Pr Y_ij _= 1|X) = *u*_*j *_+ *β*_0 _+ *β*_1_*child sex_*i *_+ *β*_2 _*maternal age_*i *_+ ... + *β*_3_*Traffic_*i *_+ *β*_4_*ILSEM*_*i *_+ *β*_5_*TLSEM_*j*_

where *u*_*j *_is a random census tract intercept. This approach was implemented using a recent version of the SAS procedure GLIMMIX [37].

It was likely that the *u*_*i *_would exhibit spatial correlation, from either the models for Gaussian or Binomial data. We modeled this spatial correlation by assigning the latitude and longitude of the population centroid of each census tract to each observation in the group *i*, and fitting an exponential spatial correlation structure.

We examined effect modification by median household income and maternal education for the association between birth weight and traffic and land use variables. We assessed precision in the difference of effects by calculating 95 percent confidence intervals around that difference [38].

## Results

### Descriptive Analyses

We calculated *mean birth weight *and the percentages of SGA, *preterm births *and a comparison group by categories of socio-demographic variables (table 1). The comparison group was selected as all term births (≥ 37 to 45 weeks) ≥ 2500 g just for the purpose of the descriptive analyses. Most of theof weight births in our study were of White mothers (75.3 percent). Mean birth weight of newborns of African-American mother was about 230 g lower than that of White; lower mean birth weight was also seen for births from mothers of Hispanic or Asian background. Because of matching in defining SGA, the percent by ethnicity of mother among SGA births were similar to those in the comparison group. The proportions of births from non-White mothers among preterm births were greater than those of the comparison group, by a range of 1.6 to 5 percent.

**Table 1:** Descriptive statistics for birth weight, small for gestational age and preterm births by socio-demographics and mother characteristics among all singleton births born in Eastern Massachusetts between 1996 and 2002

**Categorical variables**	**All births (% of total)**	**Mean birth weight (in grams) (SD)**		**Small for gestational age N (%)**		**Preterm birth N (%)**		**Comparison group (≥ 37 weeks; ≥ 2500 grams) N (%)**	
	
Mother race									
White	75.3	3463	(545)	32103	76.2	22160	66.8	266329	76.0
African-American	7.4	3236	(638)	2938	7.0	3994	12.0	24530	7.0
Asian	6.0	3241	(510)	2471	5.9	2194	6.6	20900	6.0
Hispanic	11.0	3306	(565)	4531	10.8	4730	14.3	37691	10.8
Native American	0.2	3371	(581)	66	0.2	68	0.2	537	0.2
Missing	0.1	3426	(622)	0	0	38	0.1	417	0.1
									
Mother education									
Primary and secondary (≤ 8 years)	2.6	3296	(561)	1307	3.1	1126	3.4	8073	2.3
High school (>8 – 12 years)	29.0	3338	(576)	15190	36.1	11453	34.5	96981	27.7
Some college (13 – 15 years)	39.9	3481	(533)	13354	31.7	11012	33.2	145698	41.6
College or postgraduate	22.6	3433	(562)	9118	21.7	7269	21.9	79975	22.8
(≥ 16 years)									
Missing	5.9	3333	(573)	3140	7.5	2324	7.0	19677	5.6
									
APNCU									
None	0.6	3267	(714)	251	0.6	337	1.0	1825	0.5
Inadequate	7.8	3308	(571)	4511	10.7	3147	9.5	25652	7.3
Intermediate	8.0	3487	(509)	3842	9.1	1036	3.1	28985	8.3
Appropriate	48.1	3507	(487)	20185	47.9	3263	9.8	181619	51.8
Appropriate plus	35.5	3301	(627)	13320	31.6	25401	76.5	112323	32.1
									
Mother age at birth									
<20	8.9	3246	(565)	5616	13.3	4020	12.1	28308	8.1
20 – 29	36.6	3389	(550)	16783	39.9	12007	36.2	127149	36.3
30 – 34	33.7	3465	(546)	12143	28.8	10017	30.2	121240	34.6
35 – 39	17.4	3462	(574)	6196	14.7	5762	17.4	61997	17.7
>39	3.4	3422	(600)	1371	3.3	1378	4.2	11710	3.3
									
Number of cigarettes smoked during pregnancy (per day)									
None	90.0	3438	(555)	33410	79.3	29001	87.4	320511	91.5
Any amount	9.8	3208	(558)	8655	20.6	4115	12.4	29301	8.4
Missing	0.2	3369	(648)	44	0.1	68	0.2	592	0.2
									
Number of cigarettes smoked before pregnancy (per day)									
None	83.0	3437	(556)	30580	72.6	26896	81.1	295766	84.4
Any amount	16.6	3309	(566)	11398	27.1	6120	18.4	53238	15.2
Missing	0.4	3384	(620)	131	0.3	168	0.5	1400	0.4
									
Previous infant ≥ 4000 grams									
None	98.6	3411	(556)	41851	99.4	32730	98.6	344956	98.4
Yes	0.9	3938	(546)	63	0.1	175	0.5	3626	1.0
Missing	0.5	3355	(712)	195	0.5	279	0.8	1822	0.5
									
Previous preterm birth									
None	98.6	3419	(556)	41256	98.0	31946	96.3	346553	98.9
Yes	0.9	3011	(678)	658	1.6	959	2.9	2029	0.6
Missing	0.5	3355	(712)	195	0.5	279	0.8	1822	0.5
									
Gender of infant									
Male	51.2	3473	(572)	21631	51.4	18172	54.8	178071	50.8
Female	48.8	3355	(540)	20478	48.6	15012	45.2	172333	49.2

APNCU – adequacy of prenatal care utilization; N – number; SD – standard deviation

Lower mean birth weight was observed among mothers with lower education (≤ 12 years) compared births from mothers with >12 years of educational attainment. The prevalences of mothers with high school or less educational attainment with SGA and preterm births were about one third greater than that of the comparison group (31 percent and 26 percent greater respectively).

Birth weight was lower among mothers who had received less than appropriate prenatal care. The prevalence of mothers who had received inadequate prenatal care was greater for SGA and preterm births than for comparison group. However, 76.5 percent of mothers who had preterm births had received *more than appropriate *prenatal care, twice as much as the comparison group, a suggestion of at risk pregnancies. About 9 percent of mothers had reported or were diagnosed with chronic diseases or conditions of pregnancy (chronic renal disease, lung disease, hypertension, gestational diabetes or diabetes diagnosed otherwise, uterine bleeding) – 41 percent of these had sought *appropriate *prenatal care and 48 percent *more than appropriate *prenatal care.

Younger mothers (<20 years old) had on average newborns of lower birth weight compared to births from older age groups; they were more likely to have SGA or preterm births than the comparison group (5.2 percent and 4 percent respectively). Worse birth outcomes were observed for mothers with a history of smoking during or pre-pregnancy, and among those with a previous preterm birth, although the numbers for this last one were small. Gender of infant did not make any difference on birth outcome.

We calculated mean birth weight and percent of SGA, preterm birth and comparison group by quartiles of traffic and land use variables and median household income (table 2). All adverse birth outcomes were more likely with greater traffic density, closer distance to major highways, lower percent of open space, and lower median household income.

**Table 2:** Descriptive statistics for birth weight, small for gestational age, and preterm births by quartiles of traffic variables, land use, and median household income among all singleton births born in Eastern Massachusetts between 1996 and 2002

**Continuous variables (quartiles)**	**Mean birth weight (SD)**		**Small for gestational age N (%)**		**Preterm birth N (%)**		**Comparison group (≥ 37 weeks; ≥ 2500 grams) N (%)**	
	
Cumulative traffic density/1000 (vehicles-km)								
≤ 261.0	3469	(539)	9441	22.4	7187	21.7	89628	25.6
> 261.0 – 704.6	3427	(526)	10398	24.7	8105	24.4	87756	25.0
> 704.6 – 1602.0	3398	(564)	10944	26.0	8628	26.0	86674	24.7
> 1602.0	3368	(569)	11256	26.7	9207	27.7	85785	24.5
Missing (688)	3413	(560)	70	0.2	57	0.2	561	0.2
								
Distance to primary highways (meters)								
≤ 1107	3398	(555)	11067	26.3	8460	25.5	86888	24.8
> 1107 – 2150	3403	(565)	10741	25.5	8496	25.6	87196	24.9
> 2150 – 3745	3413	(563)	10482	24.9	8422	25.4	87529	25.0
> 3750	3448	(554)	9819	23.3	7806	23.5	88791	25.3
								
% land use for recreation and conservation (open space)								
≤ 4.0	3363	(568)	11184	26.6	8905	26.8	81668	23.3
> 4.0 – 9.4	3403	(567)	10861	25.8	8711	26.3	86966	24.8
> 9.4 – 17.8	3447	(547)	9658	22.9	7182	21.6	85787	24.5
> 17.8	3458	(546)	9210	21.9	7328	22.1	87770	25.0
Missing (10470)	3319	(597)	1196	2.8	1058	3.2	8213	2.3
								
Median household income (in US $)								
≤ 46336	3303	(581)	12853	30.5	10736	32.4	83081	23.7
> 46336 – 62773	3406	(564)	10984	26.1	8405	25.3	86917	24.8
> 62773 – 78583	3467	(547)	9778	23.2	7253	21.9	89304	25.5
> 78583	3486	(526)	8494	20.2	6790	20.5	91102	26.0

N – number; SD – standard deviation

### Multivariate Analyses

We estimated the covariate adjusted associations between individual and census tract level SEM (SEM model, table 3), the associations for traffic and land use variables (exposure model, table 4), and covariate adjusted associations for SEM, traffic and land use variables (full model, table 5) and all birth outcomes. To assess confounding of the SEM and indices of physical environment by each other, and of the individual versus contextual level SEM, we also examined models selectively deleting each variable, and assessed the change in the coefficients for other variables compared to the full model. The effect of that control is presented below.

**Table 3:** Independent effects of individual and area-based socioeconomic measures (not including traffic and land use variables) in the study of singleton births in Eastern Massachusetts between 1996 and 2002.

	**Birth weight**		**Small for gestational age**		**Preterm birth**	
**Model covariates**	**Change (in grams)**	**95% CI**	**OR**	**95% CI**	**OR**	**95% CI**
	
Mother's education						
High school or less (≤ 12 years)	-8.6	-12.9, -4.3	1.18	1.14, 1.22	1.11	1.06, 1.15
Some college (13–15 years)	Reference					
College or postgraduate (≥ 16 years)	6.0	2.1, 10.0	1.05	1.01, 1.08	1.05	1.01, 1.09
						
Mother's race						
White	Reference					
African-American	-112.9	-119.6, -106.3	*		1.06	1.00, 1.11
Asian	-193.0	-199.4, -186.7			0.77	0.73, 0.82
Hispanic	-78.0	-83.6, -72.3			1.02	0.97, 1.07
						
APNCU						
Inadequate	-53.4	-59.2, -47.6	1.26	1.21, 1.31	4.52	4.25, 4.81
Intermediate	-39.0	-44.6, -33.3	1.12	1.08, 1.17	1.91	1.76, 2.08
Appropriate	Reference					
Appropriate plus	-11.1	-14.5, -7.6	0.98	0.96, 1.01	9.05	8.67, 9.45
						
Median household income^†^	8.8	6.5, 11.2	0.92	0.91, 0.94	0.99	0.98, 1.01

APNCU – adequacy of prenatal care utilization; CI – confidence interval; OR – odds ratio; SD – standard deviation

Model controlled additionally for mother age, gestational age (for models with birth weight as the outcome), cigarette smoking during pregnancy, previous infant greater than 4000 grams, previous preterm birth, chronic or gestational conditions of mother, and year of birth.

*Race is already controlled for in the definition of "small for gestational age"

^†^The effect is for 1 SD change in the median household income by tract

**Table 4:** Independent effects of traffic and land use (not including SEM variables: education, race, APNCU, and median household income) in a study of singleton births in Eastern Massachusetts between 1996 and 2002.

	**Birth weight**		**Small for gestational age**		**Preterm birth**	
Model covariates*	**Change (in grams)**	**95% CI**	**OR**	**95% CI**	**OR**	**95% CI**
	
Cumulative traffic density	-2.0	-3.7, -0.3	1.02	1.01, 1.04	1.00	0.98, 1.01
Distance to major highways	5.7	3.4, 7.9	0.98	0.97, 0.99	1.00	0.99, 1.02
% land use for recreation and conservation (open space)	13.4	10.4, 16.5	0.96	0.95, 0.97	1.01	1.00, 1.03

APNCU – adequacy of prenatal care utilization; CI – confidence interval; OR – odds ratio; SD – standard deviation; SEM – socioeconomic measures

Model controlled additionally for race (where appropriate) mother age, gestational age (for models with birth weight as the outcome), cigarette smoking during pregnancy, previous infant greater than 4000 grams, previous preterm birth, chronic or gestational conditions of mother, and year of birth.

*The effect is for 1 SD change in distribution of log-transformed traffic exposures and 1 SD change in distribution of % open space by census tract.

**Table 5:** The effects of SEM, traffic measures and land use on birth weight, small for gestational age and preterm births in the study of singleton births in Eastern Massachusetts between 1996 and 2002.

	**Birth weight**		**Small for gestational Age**		**Preterm birth**	
**Model covariates**	**Change (in grams)**	**95% CI**	**OR**	**95% CI**	**OR**	**95% CI**
	
**Socio-demographic indicators**						
						
Mother's education						
Low (≤ 12 years)	-8.5	-12.8, -4.2	1.18	1.14, 1.22	1.11	1.06, 1.15
High (13–15 years)	Reference					
College or postgraduate (≥ 16 years)	6.0	2.0, 10.0	1.05	1.02, 1.08	1.05	1.01, 1.09
						
Mother's race						
White	Reference					
African-American	-113.1	-119.7, -106.4	*		1.06	1.00, 1.11
Asian	-192.2	-198.6, -185.8			0.77	0.73, 0.82
Hispanic	-77.7	-83.3, -72.1			1.02	0.97, 1.07
						
APNCU						
Inadequate	-53.2	-59.0, -47.4	1.26	1.21, 1.31	4.52	4.25, 4.81
Intermediate	-39.0	-44.6, -33.3	1.12	1.08, 1.17	1.91	1.76, 2.08
Appropriate	Reference					
Appropriate plus	-11.0	-14.4, -7.5	0.98	0.96, 1.01	9.05	8.67, 9.45
						
Median household income^†^	6.8	4.3, 9.3	0.93	0.92, 0.95	0.99	0.98, 1.01
						
**Measures of traffic and land use^†^**						
						
Cumulative traffic density	-0.4	-2.0, 1.3	1.02	1.00, 1.03	1.00	0.98, 1.01
Distance to major highways (meters)	3.8	1.9, 5.7	0.99	0.97, 1.00	1.00	0.98, 1.01
% land use for recreation and conservation (open space)	4.4	2.1, 6.6	0.98	0.97, 1.00	0.99	0.97, 1.00

APNCU – adequacy of prenatal care utilization; CI – confidence interval; OR – odds ratio; SD – standard deviation; SEM – socioeconomic measures

All variables in this table were included in one model. Models controlled additionally for mother age, gestational age (for models with birth weight as the outcome), cigarette smoking during pregnancy, previous infant greater than 4000 grams, previous preterm birth, chronic or gestational conditions of mother, and year of birth.

*Race is already controlled for in the definition of "small for gestational age"

^†^The effect is for 1 SD change in distribution of median household income by census tract, 1 SD change of log-transformed traffic exposures, and 1 SD change of in distribution % open space by census tract.

#### Individual level SEM

*High school or less *educational attainment of mother was associated with lower birth weight when compared to births from mothers who had *some college *education (-8.6 g; 95 percent confidence interval (CI): -12.9 g, -4.3 g). In contrast, being born from mothers with *college *and *postgraduate *education (≥ 16 years) was associated with a greater birth weight (6.0 g; 95 percent CI: 2.1 g, 10.0 g). Mothers with ≤ 12 year of educational attainment were similarly at greater risk of SGA and preterm births (table 3).

Being born of African-American, Asian or Hispanic mothers was associated with lower birth weight, when compared to births from White mothers. Risk of preterm birth was also increased for mothers of African-American background (odds ratio (OR) = 1.06; 95 percent CI: 1.00 1.11). No increased risk was seen for Hispanic mothers, while a protective effect was seen for Asian mothers (OR = 0.76; 95 percent CI: 0.73, 0.82).

Lower birth weight was observed among newborns from mothers who had been provided with less than appropriate prenatal care (for example, birth weight associated with inadequate prenatal care was -53.2 g; 95 percent CI: -59.0 g, -47.4 g), when compared to mothers who had appropriate prenatal care. A similar finding, but with a weaker effect (-11.1 g; 95 percent CI: -14.5 g, 7.6 g) was observed for mothers who had *more than appropriate *prenatal care. In this case, it is important to note that *more than appropriate *prenatal care is usually provided to mothers at risk of pregnancy complications, therefore at greater risk of poor birth outcomes. Excess risks of SGA and preterm birth were seen amongst mothers who had been provided inadequate or intermediate prenatal care. The risk for preterm birth was more than four-fold if received inadequate care; however, a 9-fold risk of preterm births was associated with receiving more than appropriate prenatal care, which as noted above suggests pregnancies at risk.

#### Contextual level SEM

Controlling for individual SEM (education, race, and prenatal care index, SEM model), census tract level median household income was positively associated with birth weight (8.8 g; 95 percent CI: 6.5 g, 11.2 g), inversely related to risk of SGA (OR = 0.92; 95 percent CI: 0.91, 0.94), but not associated with risk of preterm birth (OR = 0.99; 95 percent CI: 0.98, 1.01) (table 3).

When race and maternal education were excluded from the SEM model, the effect of median household income on birth weight increased to 22.4 g (95 percent CI: 19.5 g, 25.3 g), and if race but not maternal education were included, the effect of median household income was 9.4 g (95 percent CI: 7.1 g, 11.8 g). Hence, the effect of median household income was reduced by 61 percent after controlling for individual SEM covariates.

#### Measures of traffic and land use

The results for measures of traffic are presented for one standard deviation (SD) change in the distribution of the log-transformed variable, and for land use for 1 SD change in the variable distribution (table 4). The measure of cumulative traffic density in proximity to birth address was associated with lower birth weight (-2.0 g; 95 percent CI: -3.7 g, -0.3 g). An increase in birth weight was associated with increase in distance to major highways (5.7 g; 95 percent CI: 3.4 g, 7.9 g) and percent open space (13.4 g; 95% CI: 10.4 g, 16.5 g). Increased risk of SGA was associated with increase in cumulative traffic density; a reduction in risk of SGA was observed with increase in distance to roads and percent open space. No associations were seen for risk of preterm birth with indices of physical environment.

Inclusion in models of traffic and land use variables did not affect the coefficients for educational attainment, or the coefficients for race and the index of prenatal care for birth weight (full model, table 5). However, we observed a reduction in the effect of median household income on birth weight by 23 percent in the presence of indicators of physical environment (6.8 g; 95 percent CI: 4.3 g, 9.3).

The effects of indices of physical environment on birth outcomes were also reduced when SEM indicators were included in the model. The effect of distance to roads from the full model was reduced to 3.8 g (95 percent CI: 1.9 g, 5.7). When race but not educational attainment, APNCU or the indicator of median household income were included in the model that effect was reduced to 4.3 g (95 percent CI: 2.3 g, 6.2 g), while the effect from the model with educational attainment, APNCU and median household income (5.2 g; 95 percent CI: 3.0 g, 7.3 g), but not race was not very different from that of model without any of the SEM variables. The effect of land use on birth weight was also reduced in the full model (4.4 g; 95 percent CI: 2.1 g, 6.6 g), and the effect of cumulative traffic density became imprecise. The risk of SGA associated with cumulative traffic density did not change in the full model, while the protective effects of distance to roads and percent open space weakened.

There was no important difference observed for the effect of cumulative traffic density on birth weight by educational attainment (table 6). We observed differences in effects of exposures on birth weight for distance to major highways (difference: -3.1 g; 95 percent CI of difference: -6.8 g, 0.7 g), and land use as open space (difference: -5.2 g; 95 percent CI: -9.6 g, -1.0 g). In both cases, the protective effect of greater distance from highway or greater open space land in the census tract was larger in the more advantaged. For land use, the protective effect was clearly only amongst those residents of the tract with greater education. There was no difference in the effect of distance to major highways or land use by median household income. However, there was an indication of a greater effect of traffic density in the less advantaged group (difference: -2.3 g; 95 percent CI: -5.8 g, 1.2 g), although confidence interval was wide.

**Table 6:** Stratified models by level of education and median household income for the association between birth weight and traffic and land use variables in a study of singleton births in Eastern Massachusetts between 1996 and 2002

	**Educational attainment**			
	**Low (≤ 12 years)**		**High (>12 years)**	
Model covariates*	**Change (in grams)**	**95% CI**	**Change (in grams)**	**95% CI**
	
Cumulative traffic density	-0.7	-4.3, 3.0	-0.1	-1.9, 1.6
Distance to major highways	1.8	-1.3, 4.9	4.9	2.8, 7.0
% land use for recreation and conservation (open space)	0.3	-3.3, 3.8	5.5	3.1, 8.0
				
	**Median Household Income by census tract†**			
	≤ **50%**		**>50%**	
	**Change (in grams)**	**95% CI**	**Change (in grams)**	**95% CI**
	
Cumulative traffic density*	-1.7	-4.6, 1.3	0.6	-1.3, 2.5
Distance to major highways	3.8	1.0, 6.5	4.1	1.5, 6.7
% land use for recreation and conservation (openspace)	4.4	1.0, 7.9	4.2	1.3, 7.1

APNCU – adequacy of prenatal care utilization; CI – confidence interval; SD – standard deviation Models controlled additionally for race, APNCU, mother education and median household income by census tract (where necessary), mother age, gestational age, cigarette smoking during pregnancy, previous infant greater than 4000 grams, previous preterm birth, chronic or gestational conditions of mother, and year of birth.

*The effect is for 1 SD change in distribution of log-transformed traffic exposures and 1 SD change in distribution of % open space by census tract.

†Categories of median household income: below and above the median of the distribution of the variable

We also examined three way interactions between maternal education, median household income by tract, and our measures of physical environment and found no important differences (data not shown).

## Discussion

We examined the effects of social and physical environment on birth outcomes in a study of singleton births in Eastern Massachusetts, North East US, between 1996 and 2002. Two key components of this study are worth mentioning. *First*, we used a two-level hierarchical model to estimate the variability in birth outcomes due to individual and contextual level SEM and indices of physical environment. A number of epidemiologic studies from the US and other countries have shown associations between air pollution and birth weight and preterm births, in particular with carbon monoxide and particulate matter [19,21,22,24,39-41]. The evidence however has not been fully corroborated possibly due to inadequate control for individual and neighborhood level socioeconomic indicators. Factors that account for socioeconomic position of the mother have also been associated with birth outcomes: racial/ethnic background, education, and area-based factors such as income and level of poverty [20,30,40,42]. However, only a limited number of studies have examined the influence of these factors, either alone or in combination, on the effects of air pollution on birth outcomes. Several studies from California, US, have examined air pollution effects on birth weight and preterm births controlling for individual or area-based SEM, but rarely controlled for both or examined interaction [2,21,24]. In Wilhelm and Ritz [26], individual and neighborhood-level SEM measures were adjusted simultaneously, however, using a one-level model. A recent study of the same population examined confounding and effect modification of traffic effect estimates for preterm birth by both individual and contextual SEM variables in two-level random effect models [43]. In a hierarchical multilevel model, Williams and coworkers [42] also examined relations between air pollution and SEM variables in multi-level models, although they did not focus on traffic pollution specifically.

Using the two-level hierarchical model, we found effects of individual and contextual level SEM for all three outcomes in the study (birth weight, SGA, and preterm births); the effects of traffic and land use variables were mainly seen with birth weight, with the exception of an effect of cumulative traffic density on SGA. We found that mothers with lower educational attainment were more likely of adverse births than mothers who had higher education. Lower birth weight was seen among African-American, Asian and Hispanic mothers when compared to births from White mothers; risk of preterm birth was greater for mothers of African-American background than for any other ethnic group. Having had less than appropriate prenatal care also increased the risk of adverse birth outcomes. None of the effects of individual SEM were affected when controlling for the contextual level SEM or indices of physical environment. Mothers from neighborhoods with lower median household income were also at greater risk of adverse birth outcomes. Measures of traffic exposure, estimated at the residential address of mother at time of birth, were associated with lower birth weight, and for cumulative traffic density with SGA, after control for both individual and area-based SEM.

The two-level modeling approach enabled us to control for confounding of one factor by the other: individual and contextual SEM, and physical environment. While the effect for educational attainment, race and index of adequacy of prenatal care on birth weight did not change after controlling for indicators of physical environment (traffic and land use), the coefficient for median household income was reduced by 23 percent. We also saw a reduction in the effects of traffic and land use variables on birth weight after controlling for SEM. The presence of *race/ethnicity *of mother in SEM model reduced the effect of median household income on birth weight by 58 percent. Including just race/ethnicity in exposure model reduced the effect on birth weight of cumulative traffic density by 62 percent, distance to major roads by 25 percent, and that of land use as open space by 47 percent. These results suggest that *race/ethnicity *of mother is a strong confounder in this study. Racial/ethnic inequalities do predict health disparities, and such inequalities are a representation of a history of racial discrimination, economic deprivation, and the greater likelihood of adverse exposures throughout life [15,44]. The control for both race/ethnicity and other measures of socioeconomic position was an important aspect of this study; an aspect that is considered as critical in studies of social inequality [45].

The precision of the observed relationship between air pollution and adverse birth outcomes in other studies has been largely affected by the measure of exposure assigned to each individual in the study. The majority of the studies that examined this relationship have not taken into account variation in pollution exposure within small localities, which can lead to exposure misclassification and distortion of the association. Two studies in California, US, merely averaged monitors within few miles of the residence of the child, for example [2,30]; a study in Europe [19] and another one in Brazil [40] used averaged county level data. As it has been indicated in previous studies, small scale variations are related also to variations in the socio-economic status [28]. We used individual traffic measures as predictors of air pollution, the *second *key component in our study: cumulative traffic density and distance to major highways within a radius of the subjects' home. Distance to roadways has been reported to approximate somewhat better personal exposure levels of air pollutants than ambient measurements. Studies have reported strong gradients of pollutant concentrations and particle size distribution for distances beyond 100 meters from the roads [46,47]. In our study we observed associations of traffic variables with reduced birth weight, suggestive evidence with SGA, but not with preterm births. In combination with the indicator of land use, our results suggested that the increased level of urbanization increases the likelihood of exposure and that of adverse births. We found an association of increased open space and space for recreation with a better outcome of birth weight. It may be suggested that this variable indicates greater distance to traffic, more green areas, and less urbanized residence, therefore contributing to less exposure.

The geographic differences in the distribution of air pollutants are related to the geographic distribution of wealth and socioeconomic status [18,48,49]. However, traffic can only partly explain the variability in exposures observed in diverse neighborhoods. Increased susceptibility to the effects of air pollution due to disadvantaged socioeconomic status may be related to greater likelihood for co-exposures due to poor living conditions or occupational exposures, in addition to factors like reduced access to health care, poorer nutrition, and psychological stress and violence. We examined the variability in exposure effects by categories of SEM, and found suggestive evidence that educational attainment modified the effects of indices of physical environment on birth weight, with worse outcomes among the disadvantaged. Cumulative traffic density had a greater impact on births from mothers with lower educational attainment, and the positive effects of distance to major road and land use as open space were less in the lower education group. There was an indication of a greater effect of cumulative traffic density in the lower category of median household income. Overall, we observed a signal for an interaction between measures of social and physical environment.

There are limitations to this study that need to be mentioned. Exposure was estimated by distance to major highways and cumulative traffic density at individual birth addresses, as an approximate measure to concentrations of air pollutants. These exposure measures were, however, not directly related to personal air pollutant concentrations of each study subject. Also, mother address at time of birth was used to assign individual exposure indices. That address was not necessarily the address of mother during pregnancy, and we had no information available to correct for this. Both these limitations do introduce misclassification, and due to the non-selective nature of this error we would expect our study estimates to be underestimated. We had self-reported information on smoking during and before pregnancy which we used in this study to control for potential confounding by smoking. Because of the imprecision of the self-reported measure of this factor, one can argue that there may be residual confounding biasing the study estimates. However, in this study we controlled for a number of SEM and since smoking is strongly correlated with social class, residual confounding by smoking is expected to be small.

In conclusion, the study offers a way to look at adverse health in two perspectives simultaneously: the physical and social environment. By using a broader view in trying to understand the mechanisms that determine population health, as the example in this study, one can contribute to improving epidemiologic research and hope to contribute in reducing health disparities in populations.

## Abbreviations

ADT: average daily traffic; APNCU: adequacy of prenatal care utilization; CADT: cumulative average daily traffic; MHD: Massachusetts Highway Department; SEM: measures of socioeconomic status; SGA: small for gestational age; PT: preterm; TIGER: Topologically Integrated Geographic Encoding and Reference Systems

## Competing interests

The authors declare that they have no competing interests.

## Authors' contributions

All authors have made substantial contribution to this work. AZ and JS coordinated the study. AZ analyzed the data and drafted this manuscript. SJM participated with geocoding and exposure estimation for this work. AZ and JS are the guarantors for the results and interpretation of the study findings.
